# Supplementary material for: Are carbon emissions trading and green financial instruments synergistic? -Comprehensive quantitative research based on content analysis
Source: PLoS One. 2024 Mar 7;19(3):e0298601. doi: 10.1371/journal.pone.0298601 (PMC10919601; doi:10.1371/journal.pone.0298601)
Supplement: S1 Data — (ZIP) [file pone.0298601.s001.zip › Data/地级市Z_F工作报告.zip!/╝¬┴╓/░╫│╟╩╨/└┤╫╘╣1⁄2╓┌║┼í╛┬φ┐╦╩2╛▌═°í┐_2017.docx]

各位代表：

　　现在，我代表市政府，向大会报告政府工作，请予审议，并请市政协各位委员提出意见。

　　一、本届政府工作回顾

　　市五届人大一次会议以来，我们按照省委、省政府的部署要求，在市委的正确领导下，在市人大、市政协的监督支持下，聚焦建设吉林西部生态经济区，抢抓机遇、乘势而上，攻坚破难、砥砺前行，全市经济社会事业取得重大进展，为决胜全面小康、让全市人民过上更加美好的日子奠定了坚实基础。

　　——经济发展稳中有进。坚持主动适应新常态，积极应对经济下行压力，保持经济稳定增长。预计2016年，地区生产总值完成730亿元，规模以上工业增加值完成221亿元，固定资产投资完成740亿元，地方级财政收入完成42亿元，主要经济指标增速保持全省前列。粮食生产能力跃上百亿斤台阶，畜禽饲养总量实现“四连增”。五个县（市、区）均获全国粮食生产先进县称号。能源、农产品加工、装备制造、医药等重点产业不断壮大，工业用电量增速连续三年位居全省第一。服务业增加值年均增长8.6%，旅游业收入年均增长25%。

　　——重点改革不断深化。坚持以改革破难题、增动力，由政府承担的6大领域138项重点改革有序开展，供给侧结构性改革扎实推进。“放管服”改革不断深化，清理减少行政审批项目215项，市级下放管理权限179项，基本完成事业单位改革分类任务，建立了权力清单、责任清单。商事制度改革全面实施，“五证合一”“两证整合”有效展开。农村金融综合改革试验区启动建设，我市成为全国唯一试点城市。农村改革持续深化，财税、科技、文化、教育、卫生、社保、户籍等改革稳步推进。大安市被纳入全省扩权强县试点。

　　——开发开放深度拓展。坚持走出去、请进来，以大开放促进大发展。招商引资到位资金年均增长16%，引进实施亿元以上项目714个，雏鹰400万头生猪、弘泰煤焦油轻质化等一批超50亿元重大项目落地建设。开发区平台集聚能力不断增强，入驻企业780户，金塔、风雷在“新三板”挂牌，白城工业园区被评为国家农业产业化示范基地，镇赉经济开发区荣获“中国示范园区”称号，大安市两家子化工园区成为全省超百亿元项目基地。洮南市被确定为国家现代农业示范区。嫩江湾、查干浩特晋升为国家4A级景区。通榆县、大安市成为全国电子商务进农村综合示范县。我市被确定为国家杂豆出口转型示范基地、国家出口杂粮杂豆质量安全示范区，列入第二批国家信息消费试点。民营企业突破一万户，收入突破千亿元。

　　——生态环境明显改善。坚持生态立市，启动河湖连通工程，连通水库泡塘60个，新增蓄水能力2.5亿立方米，湿地增加到750万亩。莫莫格列入国际重要湿地名录，嫩江湾、牛心套保、四海湖晋升国家湿地公园。持续开展大造林活动，完成造林205万亩，森林覆盖率达到12.5%。综合治理草原60万亩。大气、水体、土壤污染专项治理扎实开展，空气质量不断改善，地表水水质达到国家用水标准，城市饮用水源地水质达标率100%。全市降水量连续六年超过400毫米，地下水位平均上升近1米，生态效益逐渐显现。

　　——扶贫攻坚扎实推进。坚持把扶贫作为头等大事，围绕“两不愁、三保障”目标，突出精准扶贫、精准脱贫，组织实施了百项扶贫攻坚工程，完成191个贫困村整村推进，探索了光伏扶贫、电商扶贫等新模式，通榆馆成为淘宝第一个精准扶贫地方馆。在整村推进脱贫32.4万人的基础上，2016年全面开展精准脱贫攻坚，全市实现11.5万贫困人口精准脱贫。

　　——战略融入成效显著。坚持抢抓机遇、纳入国家大布局，继省委、省政府把白城确定为吉林西部生态经济区后，我市被列入全国生态保护与建设示范区、生态文明先行示范区、水生态文明建设试点城市，被批准为全国唯一风电本地消纳综合示范区、首批创建新能源示范城市、老工业基地调整改造重点规划城市。积极融入开放大通道，白阿铁路扩能改造竣工，长白快铁加快建设，平齐、通让、长西铁路改造全面开工；嫩丹高速坦途至黑水段通车，嫩江大桥开工建设；长安机场即将通航，构建了现代立体交通网络，区位边缘化的劣势得到明显改善。通榆500千伏变电站投入运行，洮南500千伏输变电工程开工建设，白城至扎鲁特旗500千伏超高压汇集送出工程全面启动，电力外送通道有望近期开通。

　　——城乡面貌大为改观。坚持推进生态城镇化建设，城镇化率达到51%。启动建设生态新区，完成了核心区“七通一平”，打造了鹤鸣湖景观，引进碧桂园、新景地等企业，新城家园入住使用，现代新城整体框架已经形成，生态新区?林海镇被列入国家新型城镇化试点。抓住列入全国首批海绵城市试点机遇，全面实施老城改造，小区变花园、老城变新城即将成为现实。统筹推进县城、重点镇和新农村建设，打造省级示范村202个、重点村87个。镇赉县荣获“中国宜居城镇”、国家级园林城称号。

　　——民生福祉稳步提升。坚持着力惠民生、增民利，持续办好民生实事。城镇新增就业18.9万人，城镇登记失业率低于3.7%。城镇和农村常住居民人均可支配收入分别达到21300元、8370元，年均增长9.8%、8.7%。各项保险待遇水平、困难群体救助标准稳步提高。建设廉租房38万平方米，改造棚户区372万平方米、“暖房子”430万平方米、农村危房10.4万户，解决15万农村居民饮水安全问题，群众生活条件不断改善。

　　——社会事业全面进步。坚持补短板、促均衡，组织实施科技、教育、卫生、文化等社会事业专项建设。五个县（市、区）通过国家科技进步考核、义务教育基本均衡发展验收。市林科院成为首批国家级专家服务基地。城乡基本公共卫生服务、文化基础设施实现全覆盖。“歌舞鹤乡?放飞梦想”文化系列活动成为国家级地方特色文化品牌。大安市被评为全国群众体育工作先进县。评选出“白城好人”296人、“吉林好人”96人，有4人荣登“中国好人榜”、1人荣获全国道德模范称号。推进“平安白城”建设，群众安全感持续提升。“六五”普法全面完成，镇赉县、大安市被评为全国法治县。信访工作得到加强和改进。安全生产工作获全省优秀等次。率先在全省完成食品药品安全示范县创建任务。夺取了2013年、2014年抗洪抢险救灾和恢复重建的重大胜利。国防教育深入开展，双拥活动扎实推进，我市实现全国双拥模范城“四连冠”，洮南市实现全国双拥模范城“六连冠”。民族宗教、外事侨务、广播电视、人口计生、妇女儿童、残疾人、老龄等工作取得新成绩，气象、人防、地震、档案、地方志、红十字会等事业实现新发展。

　　——自身建设持续加强。坚持建设人民满意政府，提升行政效能。认真开展政府系统党的群众路线教育实践活动、“三严三实”专题教育和“两学一做”学习教育，全面落实整改任务，扎实整治“四风”问题，政风建设取得实效。自觉接受人大、政协监督，建议提案办复率达到100%。依法行政深入推进。政务公开不断深化。“三公”经费支出年均下降6.5%，机构编制总量保持“零增长”。审计监督机制进一步完善。党风廉政建设和反腐败工作不断加强，政府执行力、公信力明显提升。

　　刚刚过去的2016年，我们按照市委的决策部署，注重整体推进、重点突破，狠抓生态建设、扶贫开发、经济发展、老城改造，实现了“十三五”良好开局。

　　（一）坚持以修复保护为突破口，加强生态建设。编制了生态保护与建设总体规划，启动了生态保护与建设三年行动，我市生态建设进入规划引领、系统实施的新阶段。深入实施河湖连通工程，新连通泡塘20个。开展“三年造林还湿双百万”活动，造林37万亩，修复湿地40万亩。综合治理草原26万亩。引进“桃花源”公益基金，推进向海生态保护和适度开发。实施“蓝天清水净土”工程，白城发电公司率先实现超低排放，整合集中供热企业3家，淘汰燃煤小锅炉405台，生态保护红线划定有序推进。

　　（二）坚持以产业扶贫为突破口，开展脱贫攻坚。省市县乡四级联动，实行包村包户帮扶机制，创新识贫定贫办法，搭建了精准扶贫大数据平台。多渠道筹措扶贫资金，实施产业扶贫项目1442个，有37个村被列入全国乡村旅游扶贫重点村。对因病、因学致贫采取精准扶持政策，加大社会帮扶救助力度。实施美丽乡村建设，启动危房改造、道路建设等“十大工程”，完成了通榆陆家村、镇赉四方山屯易地搬迁。

　　（三）坚持以项目建设为突破口，推动经济发展。开展早落地、早开工、早见效“三早行动”，实施亿元以上项目266个。借助台企“一会一活动”和“首届全球吉商大会”等契机，全市招商引资到位资金1130亿元。启动工业转型升级三年行动，企业利润实现正增长，产值超亿元企业达到170户。实施农业现代化总体规划，农作物播种1586万亩，调减普通玉米143万亩，畜禽饲养总量达到5400万头（只），新型农村经济合作组织发展到9280个。开展服务业发展攻坚，服务业增加值占GDP比重达到38%。

　　（四）坚持以海绵城市建设为突破口，实施老城改造。实行市区联动、全民动员，依法拆除违建14.6万平方米。实施老城改造工程和海绵城市建设项目219个，完成21条街路硬化、11条街路亮化、14条街路绿化，打造海绵小区5个，建成地下缆线管廊6.7公里，老城改造初见成效。统筹抓好新区开发，残疾人康复中心、科文中心完成主体工程。长白立交桥通车，长庆、丽江立交桥建成，连接新区与老城的通道将全面打通。启动城市智能化管理指挥中心，“智慧城市”建设取得突破性进展。

　　各位代表！

　　继往开来、接续奋斗取得的成绩来之不易，奠定了白城决战脱贫、决胜小康的坚实基础，形成了团结奋斗、聚力拼搏的可喜局面。这是在省委、省政府和市委的正确领导下，在市人大、市政协的监督支持下，全市广大干部群众共同努力的结果。在这里，我代表白城市人民政府，向全市各族人民，各民主党派、工商联、人民团体、无党派人士、离退休老同志，向驻白中省直部门和企事业单位、人民解放军指战员、武警部队官兵、公安司法干警，向关心和支持白城发展的各界朋友，致以崇高的敬意和衷心的感谢！

　　回顾几年来改革发展的艰辛历程，我们感到，工作中有差距，实践中有启示。一是必须厚植优势抓发展。目前，白城底子薄、总量小、实力弱的问题仍然突出，发展的内生动力、支撑能力不足。我们只有深化市情认识，用好优势加速释放、动能加速转换、活力持续增强的重要战略机遇期，才能走出一条落实新发展理念的新路子。二是必须增强动力促改革。制约白城发展的最大瓶颈是思想观念问题、体制机制问题。我们只有进一步解放思想、全面深化改革，才能寻求新突破、积聚新动能，在新常态下实现跨越赶超。三是必须改善民生补短板。贫困是白城全面建成小康的最大短板。我们只有既补硬短板、也补软短板，既补发展短板、也补制度短板，才能织密扎牢民生保障网，决战脱贫攻坚，决胜全面小康。四是必须依靠群众破难题。白城正处在“爬坡过坎、滚石上山”的重要关口，面临的困难多、挑战大。我们只有紧紧依靠群众，发扬“爬雪山、过草地”精神，才能跨过一道又一道沟坎，不断开创振兴发展的新局面。五是必须转变作风促落实。当前，政府职能转变还不到位，少数干部对新常态不适应、不会为、不善为，甚至存在不作为、乱作为的现象。我们只有持之以恒转职能、改作风、强服务、提效能，才能进一步做好政府工作。总结过去，是为了更好地开创未来。我们必须保持清醒坚定，在新的起点上开拓奋进，绝不辜负人民的期望和重托。

　　二、今后五年的工作

　　未来五年，决胜全面小康、让全市人民过上更加美好的日子，是摆在我们面前的重大历史任务。全市经济社会发展总体思路是：深入贯彻党的十八大以来中央重大决策部署和习近平总书记系列重要讲话精神，认真落实党的十八届三中、四中、五中、六中全会和省委十届八次全会、市六次党代会精神，坚持以“四个全面”、“五大发展”理念和“三个五”战略为引领，以推进供给侧结构性改革为主线，紧紧围绕建设吉林西部生态经济区，突出抓好生态环境、生态扶贫、生态经济、生态城市“四个重点”，以人民利益为根本、以结构调整促转型、以改革创新增动力，加快推动白城新一轮振兴发展，全面建成小康社会，努力让全市人民过上更加美好的日子！

　　我们的奋斗目标是：

　　——综合实力显著增强。地区生产总值、固定资产投资、规模以上工业总产值均突破1000亿元，增幅保持全省前列，推动白城经济高速增长，力争综合实力达到全省中等发展水平。

　　——结构调整不断优化。调优一产、做强二产、搞活三产，促进三次产业融合发展。民营经济增加值占地区生产总值比重提高到47%，高新技术产业增加值占工业增加值比重达到15%以上，形成以创新调结构、以联动促优化的发展模式。

　　——环境质量大幅提高。林地面积达到800万亩，草原、湿地均恢复到1000万亩，新增水面200万亩；城区绿化覆盖率达到38%，城市污水和垃圾无害化处理率均达到95%，主要污染物排放和能源消耗低于国家和省标准，打造天更蓝、地更绿、水更清、空气更清新的美丽白城。

　　——城乡发展更加协调。城市功能健全完善，辐射带动能力增强，完成国家级卫生城、园林城、文明城、生态城创建目标，建设生态智慧的现代城市。新型城镇化格局基本构建，城乡基础设施和公共服务体系更加健全，常住人口城镇化率达到56%，让更多群众过上舒适舒心的现代城镇生活。

　　——政府效能实现跃升。职能转变步伐加快，简政放权、放管结合、优化服务深入推进，法治政府、创新政府、廉洁政府、服务型政府建设取得明显成效，为民务实清廉蔚然成风，政府治理能力全面提升。

　　——人民生活幸福美好。城乡居民收入与经济同步增长，城镇登记失业率控制在4.5%以内。社会保障能力明显增强，各项事业繁荣发展，社会大局和谐稳定。到2018年全面完成脱贫攻坚任务，到2020年全面建成小康社会，让全市人民过上更加幸福美好的日子。

　　落实市六次党代会确定的未来五年思路目标和重点任务，我们必须牢牢把握“五个坚持”，加快建设吉林西部生态经济区，构建国家级生态经济跨省合作区。

　　（一）着力保护生态环境。突出保护优先、自然恢复为主，坚持水、湿、草、林“四位一体”，深入实施河湖连通、湿地修复、草原建设、植树造林“四大工程”，筑牢生态安全屏障，全面提升“湿地鹤乡、生态白城”形象；持续开展大气、水体、土壤污染防治“三大行动”，保护好白城的蓝天白云、碧水净土；加快构建生态文明制度体系，实现源头严防、过程严管、违法严惩，打造全国生态文明先行示范区。

　　（二）着力推进生态扶贫。坚持以生态理念推进扶贫开发，实施资源环境可承载的产业项目，支持开展生态移民工程，让贫困人口从生态建设中得到更多实惠；坚持以生态理念推进共享共建，把贫困村建设成美丽乡村，设施改造体现生态要求，环境打造融入生态元素，改善生活兼顾生态保护；坚持以生态理念推进社会治理，解决好区域性整体贫困问题，让生态文明建设成为脱贫攻坚新动力。

　　（三）着力发展生态经济。突出转型升级，发展生态农业，推行农牧结合、生态循环生产模式，建设“有机米、健康豆、精品肉、生态鱼”种养基地；发展生态工业，创建全国食源性食品安全生产基地、清洁能源开发利用示范区，建设吉林西部医药健康产业基地；发展生态旅游，打好湿地生态游、民俗风情游、冰雪体验游、乡村观光游品牌。通过推进三产深度融合、集群集聚，着力打造中蒙俄经济走廊重要的开放合作区。

　　（四）着力建设生态城市。走以人为核心的新型城镇化道路，注重集约化，突出城市规划的战略性、建设的整体性、管理的规范性，构建集约紧凑、疏密有致的现代城市格局；注重绿色化，扩大城市生态空间，提升舒适、雅致、整洁的城市景观风貌，构建绿色生产方式、生活方式和消费模式；注重一体化，实现县县通高速，迈入快铁时代，开启空中通道，着力打造区域现代化中心城市。

　　坚持生态立市，推进白城新一轮振兴发展，必须突出“三个关键”：一是抓好改革源动力。以改革的办法，突破路径依赖，破解制约瓶颈，激发市场和社会活力；以开放的视野，深度融入“一带一路”和“长吉图”战略，谋划推进东北生态经济跨省合作区，提升发展层次和水平。二是打造环境软实力。改进政府服务，构建“亲”“清”新型政商关系，靠良好的法治环境、金融环境、信用环境留住企业；完善基础设施，增强承载功能，靠集约便利的设施条件吸引投资；打好扶贫牌、振兴牌、生态牌，谋划国家和省级概念，靠独特的政策优势吸引项目和资金。三是强化人才支撑力。深入落实“人才兴市”战略，营造创新创业的良好氛围，让各类人才育得出、引得来、留得住，都能够有条件、有机会建功立业。路在人走，业在人创。今天的白城，正在发生日新月异的可喜变化。展望未来，我们信心满怀。只要我们上下同心、干群合力，建设生态优良、绿色文明的美丽白城，繁荣发展、包容开放的现代白城，宜居宜业、和谐平安的幸福白城，一定会成为美好现实。

　　三、今年的主要任务

　　今年是新一届政府的开局之年。政府工作总的要求是：贯彻落实中央和省经济工作会议精神，按照市六次党代会总体部署，坚持稳中求进工作总基调，以提高发展质量和效益为中心，以推进供给侧结构性改革为主线，围绕建设吉林西部生态经济区，继续狠抓生态建设、扶贫开发、经济发展、老城改造，全面做好稳增长、促改革、调结构、惠民生、防风险各项工作，解放思想、埋头苦干、奋力攻坚，推动全市经济社会发展取得新的更大突破，以优异成绩迎接省十一次党代会和党的十九大胜利召开。

　　主要预期目标是：地区生产总值增长7.5%；地方级财政收入增长5.5%；固定资产投资增长11%；规模以上工业增加值增长8.3%；社会消费品零售总额增长8.5%；城乡居民收入与经济同步增长；城镇新增就业4.2万人，城镇登记失业率控制在4.5%以内；节能减排指标完成省下达任务。

　　重点抓好以下十个方面工作：

　　（一）全力抓生态建设。以生态保护与建设三年行动为抓手，着力构建全方位、立体式的大生态建设格局。一是加快河湖连通。连通一期工程剩余的64个泡塘，新增蓄水能力3亿立方米。谋划河湖连通二期和地下水库建设，构建区域地表水与地下水循环互补体系。二是推进造林还湿。重点抓好河湖连通沿线、市区和城镇村屯周边的造林绿化，确保造林37万亩，修复湿地35万亩。完成向海生态移民。三是加强草原建设。推进蒙草生态合作项目，每个县（市、区）建设一个1000亩以上集中连片人工种草示范基地，每年全市综合治理草原100万亩以上。四是治理盐碱地。实施以燕麦种植为重点的生态修复示范工程，启动盐碱地生物修复治理项目，争取用五年时间使盐碱地得到基本治理。五是健全生态制度。启动实行“河长制”，争取生态环境损害赔偿改革试点，探索生态保护预警、污染防治联动机制，完善生态考核评价体系。划定生态保护红线，严格落实管控措施。加大退耕还林还草还湿力度，全面推行封山育林，坚决禁止林木商业采伐，严厉打击侵占草原违法行为，限期恢复草原属性和面貌，依法保护生态建设成果。

　　（二）全力抓脱贫攻坚。整合优势资源和力量，加大扶贫精度和力度，必保6.6万贫困人口脱贫。一是狠抓产业升级。推广“合作社＋贫困户”、“龙头企业＋贫困户”模式，推进光伏扶贫、庭院经济、“抗旱水源井＋柴改电”、电商扶贫等项目叠加，加快在384个贫困村发展光伏分布式电站。引导同类型、同质化项目整合，形成产业规模。二是强化政策落实。谋划推出一批基础设施、公共服务项目，争取纳入全省“1＋N”行业扶贫计划。有针对性出台一批扶贫政策，着力抓好健康扶贫、教育扶贫。鼓励支持大学毕业生、返乡农民工和外来投资者创业兴业，带动脱贫致富。整合涉农资金，落实开行、农发行基础设施扶贫资金，必保每个贫困村资金规模达到700万元。三是建设美丽乡村。围绕改善人居环境，突出水、电、路、房等基础设施，全面推进“十大工程”，确保建档立卡贫困户危房改造任务全部完成，力争用两年时间把贫困村全部建成新农村。四是加强督查考核。完善资金监管、调度督查、考核评价机制。充分发挥大数据平台作用，实行台账管理，严格标准程序，做到精准扶贫、精准脱贫。

　　（三）全力抓项目投资。开展项目建设攻坚年活动，以项目大突破注入发展新动能。一是抓开工。继续组织实施“三早行动”和项目集中巡检，全年实施超5000万元项目不少于200个，切实发挥有效投资的关键作用。各县（市、区）必保新建超亿元工业项目2个以上，工业园区、经开区必保3个以上，工业项目投资占比60%以上。推进嫩丹高速黑水至边昭段、500千伏超高压汇集送出等工程，确保长安机场、长白快铁投入运营。二是抓谋划。立足资源禀赋、产业基础，重点围绕清洁能源开发和特色农产品、规模畜产品、水产品精深加工，分门别类编制产业和项目规划。积极谋划进京高铁、交流特高压等重大基础设施项目。全年谋划超亿元项目130个以上，达到项目建议书程度。三是抓招商。强化“产业树”理念，开展补链招商、专题招商，改“碰啥招啥”为“缺啥找啥”，着力打造全产业链条。拓展吉商大会成果，推进与中国华信、海油机械、国药集团等大企业洽谈对接，搞好跟踪服务，力促项目签约落地。争取全年招商引资到位资金1300亿元，增长15%。

　　（四）全力抓工业转型。以扩总量、调结构、上水平为核心，推进工业转型升级三年行动，力争工业总产值突破800亿元。一是实施小微企业生成计划。落实发展民营经济、支持小微企业政策措施，加快构建融资服务、创业服务、市场服务、创新服务、人才服务体系，搭建“众创、众包、众扶、众筹”支撑平台，推动“政银企保”对接，促进新企业生成、小企业成长，力争新发展小微企业200户以上。二是实施重点企业培育计划。坚持“一企一策”，围绕解决要素供给、市场开拓、管理提升等问题，开展包保帮扶，加快振兴实体经济。从今年开始，力争用三年时间培育产值超100亿元企业1户、超50亿元3户、超10亿元30户、超亿元300户。今年必保规上工业企业突破330户。抓住国家支持贫困地区企业上市契机，争取每个国贫县三年内至少新增2户上市企业。三是实施重点产业跃升计划。突出抓好能源、农产品加工、装备制造、医药健康“两支两优”产业。启动光伏“领跑者”基地、清洁能源储能消纳示范园区建设，引进冶金、锻造等现代高载能项目，推进清洁供暖，开展区域性直购电试点，形成电价洼地，打造全省最大的清洁能源基地；加快推进雏鹰400万头生猪、吉西300万只肉羊、飞鹤50万只奶山羊、首农2.5万头奶牛，开工建设牧原300万头生猪、阳春羊奶150万只奶山羊等全产业链项目，打造“生态牧业基地”；抓好中一精锻、永固连杆等企业技改扩能，打造精益管理样板，逐步向“智慧工业”升级；加快建设医药健康产业园、中加燕麦产业园，培育大健康产业。四是强化园区平台建设。研究制定各类开发区发展战略规划，明确功能定位，突出特色产业，探索差异化发展道路。加快完善各类开发区配套功能，创新开发区行政管理体制，合理规划建设用地，清理盘活闲置、低效用地，提高投入产出比。

　　（五）全力抓农业发展。以农业产业化为抓手，发展现代农牧渔业，促进三产深度融合。一是突出种养结合。扩大“粮改饲”试点，规模种植燕麦、苜蓿等饲草，发展舍饲牧业；推广稻、苇、鱼、蟹立体养殖技术，探索种养结合型循环农业发展模式。二是突出特色发展。抓好燕麦、绿豆、辣椒、高粱等特色种植基地建设，力争扩大到500万亩。水田面积达到260万亩，争创全省水田第一大市。大力扶持棚膜经济，新建大棚2000亩。启动中药材规范化种植基地项目。三是突出规模经营。借助种植结构调整和农村金融综合改革，发挥益海嘉里、金塔集团等龙头企业带动作用，新发展农民专业合作社220家、家庭农场140家，推进土地流转190万亩。力争农业综合机械化率达到76%，规模养殖比重达到48%。四是突出品牌创建。实施增品种、提品质、创品牌“三品”战略，培育“弱碱大米”“绿色渔业”品牌。推介“白城燕麦”“白城绿豆”等地标品牌，依托杂粮杂豆国检中心，全力打造“东方燕麦之都”“中国特色杂粮杂豆之城”。五是突出设施建设。加快月亮泡蓄滞洪区建设、嫩江干流治理、灌区续建配套改造，推进节水灌溉工程建设，完善小型农田水利设施，整理高标准基本农田23万亩。

　　（六）全力抓服务业提升。打好服务业发展攻坚战，力争服务业增加值增长8%以上。一是发展商贸流通。优化商业网点布局，改造提升农贸市场。支持传统商贸企业转型升级，开展促销专项行动，推动欧亚商都、宏达农机等企业做大做强。加快国家杂豆出口转型示范基地建设，促进外贸回稳向好。二是发展现代物流。谋划建设绿色农产品物流园、杂粮杂豆集散中心，加快推进内陆港二期、港原建材城等项目。做大电子商务产业园，力争实现电商服务站行政村全覆盖。三是发展生态旅游。大力开发湿地生态、草原风光、冰雪体验、农业休闲等旅游产品，规划自驾游、骑行游、科考游等新项目。抓好查干浩特滑雪场、嫩江湾修复保护等重点项目，推进通榆至向海、洮南至向海、莫莫格至月亮泡旅游公路建设，谋划建设五星级宾馆。力争旅游收入突破70亿元。四是发展房地产业。培育住房租赁市场，引导企业利用存量房发展旅游、养老、创业等地产。落实房地产交易税收优惠政策，推广“农民安家贷”，鼓励农民进城购房。此外，改造提升传统服务业，鼓励发展健康养老、文化体育等新兴业态。

　　（七）全力抓老城改造。以海绵城市、管廊城市试点为契机，坚持产城互动、建管并重，加快推进城镇化。一是完成老城改造。全面完成44条街路、152个小区、10个标准化社区、40座水冲公厕、6个公园广场、5个城市出入口和步行街、地下管廊改造建设，同步做好绿化、美化、亮化、净化、智能化工作，夺取老城改造决定性胜利。必保海绵城市试点通过国家考核验收。二是加快新区开发。完善基础设施，确保长庆、丽江立交桥通车。配套公共服务，完成科文中心布展，推动残疾人康复中心、学校投入使用。加快产城融合，开工建设商业综合体、健康产业园等项目。三是打造特色城镇。启动新一轮县城建设规划修编，抓好特色县城建设。加快推动县域经济转型升级，确保整体或单项在全省排位大幅前移。加强小城镇公共设施建设，推广通榆陆家村、洮北中兴村经验，每个县（市、区）至少打造2个特色小镇，探索新型农村社区建设模式。四是加强城市管理。严格落实市容和环境卫生管理条例，全面启动“四城联创”，推进城市执法体制改革，探索建立信息化管理模式，加大市容市貌、交通秩序、车辆停放等整治力度，开展“告别不文明行为”活动，制定物业考核体系，让我们的城市更加整洁、更加有序、更加文明。

　　（八）全力抓深化改革。以供给侧结构性改革为主线，统筹推进重点领域改革。一是推进“三去一降一补”。建立去产能公示制度，加大处置“僵尸企业”力度。消化商品房库存82万平方米，努力提高棚改货币化安置比例。支持企业市场化、法治化债转股，降低企业负债率。深入开展清费减负行动，助力企业降成本、增效益。重点补齐生态脆弱、农村贫困、经济发展后劲不足、城乡建设相对滞后等短板。二是推进农业供给侧结构性改革。尊重市场规律，适度调整种植结构，发展特色种养业、林果业、水产业基地，扩大绿色优质农产品有效供给。推进农业标准化示范区建设，通过无规定动物疫病区国家验收，提升农畜产品品质。推进农村土地“三权分置”并行改革，完成农村土地确权颁证工作。全面启动农垦改革、供销社综合改革，搞好粮食收储制度改革，完成国有林场改革任务。实施农民增收行动计划，拓展经营性、工资性和财产性收入渠道。三是推进农村金融综合改革。围绕建设国家级农村金融综合改革试验区，开展金融产品创新，提升金融服务可获得性、便利性，降低融资成本。健全农村综合产权交易市场，推进农村承包土地经营权和农民住房财产权抵押贷款试点，扩大土地收益保证贷款投放领域和规模。发起设立现代农业发展基金，加大对燕麦等重点产业和发展领域的支持力度。开展农村信用合作试点，完善融资担保服务体系，积极申请农业专业保险牌照，成立农村金融投资公司，实现村级金融服务站全覆盖。四是推进国资国企改革。成立国资管理机构，加强国有企业、国有资源资产和非经营性资产集中统一监管，突出主业、剥离辅助，搭建投融资平台，确保国有资产保值增值。完善公司法人治理结构，公开聘用职业经理人，加快建立现代企业制度。此外，统筹推进财税体制、科技创新、医药卫生等领域改革。

　　（九）全力抓民生事业。加快补齐民生短板，让群众生活有质的提高。一是办好民生实事。主要是10个方面：（1）启动创业就业培训计划，实现城镇零就业家庭援助率100%，创建省级高质量就业示范行政村10个。（2）开展全民参保登记计划，加快推动城居保与新农合整合。（3）改造棚户区9290套，抓好农村危房、农村厕所改造。（4）实施二水厂改造达标、三水厂扩能增量，完成污水处理厂提标改造；巩固提升1万名农村群众饮水安全保障能力。（5）加密市区公交班次，提升公交覆盖面和服务水平；建设农村公路200公里，加强农村公路养护管理。（6）规划建设体育馆、游泳馆、滑冰馆，维修改造市体育场、市文化中心，建设村级综合性文化服务中心147个。（7）建设居家养老服务平台，开展老年人意外伤害保险工作。（8）改善义务教育薄弱学校基本办学条件，新建公办幼儿园3所。（9）开展扶老助残救孤济困行动，城乡低保标准分别达到上年收入的20%、30%。（10）完成集中供热企业整合，淘汰所有燃煤小锅炉，结束市区分散燃煤供热历史。二是发展社会事业。加强创新型人才、企业家队伍建设，加快培育一批科技型骨干企业。突出教育优先发展战略，加大教育投入力度，强化校园安全管理，深化职业教育产教融合、校企合作。注重培育生态文化，完善文化体育设施，持续打造“歌舞鹤乡?放飞梦想”和“快乐运动?幸福鹤乡”群众文体活动品牌，建设文化白城。全面推开公立医院综合改革，构建更高水平的城乡医疗卫生服务体系。加强计划生育服务管理。深入开展“德润鹤乡?感动白城”“全民志愿?公益白城”主题活动。加强国防后备力量建设，做好国防和军队改革服务工作，推动军民融合深度发展。保障妇女儿童权益，支持慈善事业发展，做好民族宗教、外事侨务、档案、地方志等工作。三是加强社会治理。创新基层治理模式，推进“互联网＋社区服务”省级试点。完善立体化治安防控体系，实现视频监控系统城区全覆盖。落实安全生产领域改革发展意见，推进安全生产“四化融合”。依法规范信访工作。全面实施“七五”普法规划。深化食品放心工程，争创“全国食品安全示范市”。抓好气象服务、抗旱防汛、防灾减灾等工作。

　　（十）全力抓职能转变。以转职能为核心，以提效能为重点，着力建设廉洁高效、人民满意的政府。一是更新观念。开展“解放思想大讨论、推动发展大跨越”主题教育实践活动，抓住我省与浙江省对口合作契机，学习借鉴发达地区先进经验，从思想观念、体制机制、方式方法等方面全方位对标，务求政府理念行为发生积极变化。二是优化软环境。推进“放管服”改革，抓好行政审批标准化建设，开展减证便民专项行动，探索实施“多证合一、一照一码”，全面推开“两随机、一公开”监管，加快政务服务“一张网”建设。集中开展软环境整治行动，选聘一批民营企业家担任软环境监督员。加强“诚信白城”建设，健全守信践诺、失信惩戒机制。三是依法行政。制定法治政府建设实施方案，把依法行政纳入绩效考评。完善重大行政决策程序，注重决策风险评估，增强公众参与实效。高度重视立法工作，自觉接受人大、政协监督，认真办理人大代表建议和政协提案。深入推进政务公开，及时回应社会关切。四是廉政勤政。加强政府系统廉政建设，完善不想腐、不能腐、不敢腐的长效机制。加大行政监察力度，推进审计全覆盖。健全正向激励机制，强化责任意识、攻坚意识、争先意识，做到敢担当、能担当、善担当，形成崇尚实干、狠抓落实的施政新风。

　　各位代表！

　　新的征程已经开启，新的目标催人奋进。让我们紧密团结在以习近平同志为核心的党中央周围，在省委、省政府和市委的正确领导下，在市人大、市政协的监督支持下，同心同德，奋力攻坚，践行新发展理念，决胜全面小康，为全市人民过上更加美好的日子而努力奋斗！
